# Mindfulness-Based Student Training Improves Vascular Variability Associated With Sustained Reductions in Physiological Stress Response

**DOI:** 10.3389/fpubh.2022.863671

**Published:** 2022-07-18

**Authors:** Andreas Voss, Martin Bogdanski, Mario Walther, Bernd Langohr, Reyk Albrecht, Georg Seifert, Mike Sandbothe

**Affiliations:** ^1^Institute of Innovative Health Technologies (IGHT), Ernst-Abbe-Hochschule Jena, Jena, Germany; ^2^Institute of Biomedical Engineering and Informatics (BMTI), Technische Universität Ilmenau, Ilmenau, Germany; ^3^Department of Pediatric Oncology and Hematology, Charité—Universitätsmedizin Berlin, Corporate Member of Freie Universität Berlin and Humboldt-Universität zu Berlin, Berlin, Germany; ^4^Department of Basic Sciences, Ernst-Abbe-Hochschule Jena, Jena, Germany; ^5^Jena Achtsamkeit, Jena, Germany; ^6^Department of Social and Behavioral Sciences and Department of Medicine, Friedrich-Schiller-University Jena, Jena, Germany; ^7^Departamento de Pediatria, Faculdade de Medicina, Instituto de Tratamento Do Câncer Infatil (ITACI) Universidade de São Paulo, São Paulo, Brazil; ^8^Department of Social Work, Ernst-Abbe-Hochschule Jena, Jena, Germany

**Keywords:** mindfulness-based stress reduction, mindfulness-based interventions, autonomic regulation, pulse wave variability, heart rate variability, non-linear dynamics, higher education, University students

## Abstract

In today's fast-paced society, chronic stress has become an increasing problem, as it can lead to psycho-physiological health problems. University students are also faced with stress due to the demands of many courses and exams. The positive effects of mindfulness-based stress reduction (MBSR) on stress management and self-regulation have already been studied. We have developed a new mindfulness intervention tailored for students—the Mindfulness-Based Student Training (MBST). In this study, we present longitudinal results of the MBST evaluation. Biosignal analysis methods, including pulse wave variability (PWV), heart rate variability, and respiratory activity, were used to assess participants' state of autonomic regulation during the 12-week intervention and at follow-up. The progress of the intervention group (IGR, *N* = 31) up to 3 months after the end of MBST was compared with that of a control group (CON*, N* = 34). In addition, the long-term effect for IGR up to 1 year after intervention was examined. The analysis showed significant positive changes in PWV exclusively for IGR. This positive effect, particularly on vascular function, persists 1 year after the end of MBST. These results suggest a physiologically reduced stress level in MBST participants and a beneficial preventive health care program for University students.

## Introduction

The broad field of stress research—including the study of stress factors (stressors), physiological and psychological effects, and strategies for coping with stress—has received increasing attention over many years. Inadequate stress management can lead to chronic stress, which is considered a major risk factor for physical and mental illness (1–5). This involves impairment of the immune system (6) and promotion of inflammatory processes (7) as well as higher risk for cardiovascular diseases (CVD) (8–10). The relation of mental disorders from inadequate stress management have already been examined for anxiety, depression, and burnout (11, 12). There is also consent that stress-related impairments often connects both aspects, the physiological and psychological level (13, 14). Recent studies investigated the mechanisms and consequences of occupational stress that leads to burnout (15–17). The new revision of the World Health Organization on burnout as “an occupational phenomenon resulting from chronic workplace stress” in the 11th Revision of the International Classification of Diseases (ICD-11) (18, 19) underlines this important field of research. This also applies to students in University as the campus is their workplace. Since, as shown, stress is seen as a relevant risk factor to cause or promote different types of diseases, there is also a need to strengthen the health literacy of University students. A link between perceived stress and quality of live was shown by Ribeiro, Pereira (20). In their review they found a “negative association between stress and QoL in University students, through the deterioration of various aspects related to physical and mental health.” In another study Keech, Hagger (21) suggest interventions for active stress management which might also improve student's academic performance. Therefore, we were encouraged in our commitment of developing and evaluating our novel Mindfulness-Based Student Training (MBST) program to enforce the student's stress management and coping, quality of life and health prevention.

Based on classical elements of Mindfulness-based Stress Reduction (MBSR), originally introduced by Jon Kabat-Zinn (22), we had developed and established a new mindfulness intervention for students at University. The additions and modifications to the MBSR methods were especially tailored to the students' needs in everyday life. The mediated training was intended, among other things, to strengthen stress management as well as self- and emotion regulation and thus to contribute to a personal health benefit. This approach should be accompanied by scientific examinations based on physiological biomarkers. The effects of MBSR have been the subject of numerous studies. However, in the majority of evaluations this was assessed by self-reporting measures (23–26) and only a minority used biomarkers (27, 28) or biosignal analysis (29–31). Most studies that included biosignal measurements were limited in their variety of assessment methods. They considered only a limited selection of single heart rate variability (HRV) characteristics or non-continuously derived biosignals (such as discrete values of systolic blood pressure). In our evaluation study, we aimed for a broader but also more detailed characterization that includes variability features from multiple biosignals and is represented by different domains of biosignal analysis (e.g., time, frequency, and non-linear dynamics). Repeated measurements of electrocardiogram (ECG), finger pulse photoplethysmogram (PPG, derived by pulse oximetry), and breathing activity (RESP) from the MBST intervention group and a passive control group provided information about the condition of participant's autonomic regulation. Several indices of heart rate variability (HRV), pulse wave variability (PWV), and RESP characterizing autonomic functions in different domains of biosignal analysis had been extracted and statistically analyzed. Based on this, a significant effect in terms of reduced vascular regulation (specific systolic PWV estimates) was found in our first preliminary study (32). This improvement was observed exclusively in MBST participants and demonstrated a positive health benefit in an initial examination after 8 weeks of intervention. The presented second part of this study aimed to investigate longitudinally to what extent stress reduction can be demonstrated after the complete 12-week MBST intervention. Therefore, we were now interested in examining what effect could be demonstrated at the end of the 12-week intervention and how it compared to controls up to 3 months into follow-up. In addition, we wanted to examine long-term effects in the intervention group over multiple measurement periods up to 1 year after completion of the MBST.

## Materials and Methods

In our previous study (32), the preliminary effects of the MBST program from pre to 8 weeks after starting the 12-week intervention for University students has been evaluated. The training's impact was measured in terms of HRV, PWV and RESP, and compared to a passive control group. Now the findings of the entire study with measurements until 1-year follow-up after end of MBST will be presented.

### Study Design

All procedures performed in this study involving human participants were approved by the Institutional Ethics Commission of the University Hospital Jena (4509-08/15), and in accordance with the 1964 Helsinki declaration and its later amendments. Informed consent was obtained from all individual participants included in the study.

The basic study design can be described as a (restricted randomized) controlled trial comparing two groups over time. This evaluation study included the comparison of the intervention group (IGR) and a (passive waitlist) control group (CON) at four measurement times until 3 months after end of MBST. Additionally, the follow up of the MBST participants until 1 year after end of MBST was assessed with a fifth and sixth measurement. More details on the measurement times are shown in Table 1.

**Table 1:** Measurement points with description.

**Measurement**	**Description**	**Comment**
M0	Acclimation measurement	–
M1	Before MBST	At semester start
M2	After 8 weeks of MBST	During semester
M3	After end (12 weeks) of MBST	Near semester end, at start of exams
M4	3 months after end of MBST, study end for CON	After summer break, at start of next semester
M5	6 months after end of MBST	Near semester end, at start of exams
M6	1 year after end of MBST, study end for IGR	Near semester end, but before exams

We recorded attendance at the weekly courses *via* study certificates, which acted as proof for the participants that they had passed the course for their student record book. A maximum of one absence was allowed over the 12 weeks of the course. In addition, participants kept a Mindfulness Diary, which recorded, among other things, their personally spent practice time at home. Of course, a real control of these self-reported statements could not take place. In the follow-up, after the end of the MBST course, the participants were asked about their further practice *via* several online surveys.

### Participants

At the beginning of the study, 40 participants for each of both groups were recruited. For the comparison up to the fourth measurement, involving the 12-week MBST and 3 months follow up, 31 participants in IGR and 34 in CON finished the data acquisition. This provided the basis for the statistical comparison of the two progressions. In addition, 22 IGR participants completed the final M6 measurement to enable insight into the long-term effects of the intervention. Due to dropouts, the number of participants for the final analysis is less than those recruited at the beginning. More information about the groups and their compositions is given in Table 2.

**Table 2:** Number, sex, and age of study participants per group.

**Group**	***N***	**Sex**	**Age**
**(measurements)**	**[participants]**	**[female/male]**	**[mean ±sd, years]**
IGR (M1 to M4)	31	28/3	24.4 ± 3.8
IGR (M1 to M6)	22	20/2	24.6 ± 3.6
CON (M1 to M4)	34	31/3	24.2 ± 7.2

The recruitment at the campus was directed to full-time students at the local universities (Ernst-Abbe-Hochschule Jena and Friedrich Schiller University Jena) and preferred early semesters to ensure a long participation in this study. In addition to these eligibility requirements, the following exclusion criteria were defined: Pregnancy, advanced experience with mindfulness interventions, (semi)professional athletes, diabetes, hypertension, or known heart disease. The enrollment followed a first come, first serve process. Finally, most of the participants were students of social sciences, but also of economics and engineering. In a preparatory meeting, the participants were subjected to a familiarization measurement, among other things, to become familiar with the measurement situation.

### MBST Intervention

Based on classical elements of Mindfulness-based Stress Reduction (MBSR), originally introduced by Jon Kabat-Zinn (22), we developed and established a new mindfulness intervention for students at University. The main reason for changing the authorized MBSR curriculum (https://lotheijke.com/wp-content/uploads/2020/11/8-week-mbsr-authorized-curriculum-guide-2017.pdf) was the goal to offer a course with a time structure that meets the standards of academic seminars in Germany (90 min per week in a semester), so that students can receive credit points for it across universities and faculties. In addition, it has been shown that the duration of the MBSR exercises (approximately 2.5 to 3.5 h for weekly session), while useful in the clinical context of MBSR as well as in outpatient MBSR courses, is too long for students in an academic setting. The same is true for the mindfulness day.

The resulting changes in the temporal structure of the curriculum, as well as the need to directly address students' specific issues in the course and to reduce the number of medical inputs, have led to a significant change in the course format, as expressed by the new name (MBST). This is at the same time a response to the following request found in the authorized Curriculum Guide for MBSR: “Currently, there are a wide-range of mindfulness-based programs that have developed out of the basic structure and format of MBSR. We applaud these adaptations and experiments while strongly urging our colleagues to call what they do MBSR only if they adhere to the structure and standards described herein.” (https://lotheijke.com/wp-content/uploads/2020/11/8-week-mbsr-authorized-curriculum-guide-2017.pdf, p. 1).

The 12-week MBST intervention includes weekly 90-min sessions and a 5-h “mindfulness retreat day.” In addition, there is an introductory and final session at the beginning and end. The practices taught consist of informal and formal exercises (such as body scan, different meditations, and mindful yoga) guided by MBSR trainers. Among other things, the training provided was to strengthen stress management as well as self- and emotion regulation, thereby contributing to personal health benefits and improve quality of life.

### Data Acquisition & Pre-processing

All measurements took place in a quiet and exclusive room at the campus. While sitting comfortably on an office chair the participants were asked to not talk or move during the recording. The application of the measurement equipment and verification of the signals was carried out professionally by the investigating researcher. After coming to rest, about 12 min were recorded of which 10 min were later used for analysis. At each measurement, notes on (medical) status and recent activities or changes were recorded to capture significant changes in eligibility criteria during the study period.

The acquisition and processing of the measured biosignals included derivations from ECG, PPG, and breathing activity *via* respiration belt. Beat-to-beat intervals (RR time series) from detected R-peaks in the ECG provided later HRV analysis. Analogous to the systogram and diastogram from blood pressure, the amplitude series from detected systolic maxima (PSYS) and diastolic minima (PDIA) from the PPG were linearly transformed and then allowed PWV analysis. The maxima and minima in the RESP signal resulted in inspiration (RESPin) and expiration (RESPex) time series, respectively. Finally, the RR as well as the PSYS and PDIA series were subjected to an adaptive filtering which corrected ectopic beats and artifacts that could affect further analysis (RR intervals then denoted as NN/normal-to-normal intervals). These interval and amplitude series formed the basis for all further feature calculations. Representative PSYS series showing typical examples of vascular variability for both groups at different measurements are provided in the Supplementary Materials (Supplementary Figure 1).

More details about the study design, participants recruitment and grouping, an MBST description and curriculum as well as further explanations to data acquisition and pre-processing have already been given in the first publication.

### Feature Extraction

The preselection of measured variables for the preliminary feature set of the following statistical analysis was done with regard to the findings of our prior study and to the most common standard features of variability indices in time and frequency domains as suggested by the Task Force of the European Society of Cardiology and the North American Society of Pacing and Electrophysiology (32, 33). These include (among others) mean values and measures of dispersion (e.g., meanNN and sdNN), proportional measures of specific values (in this case pNN50), the power in specific frequency bands (such as low or high frequency powers, and their ratio LF/HF), but also features of symbolic and non-linear dynamics.

In the present analysis some additional and modified features were included. These are the SDA1 and RMSSD of the amplitude variabilities in the PSYS and PDIA series. Analogous to the HRV counterparts, these variables give a measure of the standard deviation of the averages of amplitude series in all 1-min segments (_sdaAMP1 with prefixed PSYS or PDIA) and the square root of the mean squared differences of successive amplitudes (_rmssd with prefixed PSYS or PDIA). Another change was made to the estimates given by the high-resolution joint symbolic dynamics (HRJSD). Instead of specific single patterns quantifying non-linear interactions in the given series, a more summarized approach by using the Rényi entropy (with α = 2) of the interactions between the beat-to-beat interval series of HRV and the amplitude series of PWV (HRJSDrenyi2_BBIPSYS or HRJSDrenyi2_BBIPDIA) as well as within all three of these series in the multivariate HRJSD (mHRJSDrenyi2) has been utilized. The Rényi entropy serves as a measure for the complexity and randomness of a time series. In this case, the method is used to quantify the complexity of the joint patterns of symbolic dynamics of two or three input series. For further details, please see the variable-list and description in the supplementary materials (Supplementary Table 1).

Using these features from different disciplines of biosignal analysis, the physiological state of the participants will be determined. This concerns in particular functions of the autonomic nervous system and the antagonistic parts of the sympathetic and parasympathetic nervous system functioning in it. Sympatho-vagal balance can provide information about physical stress levels but also about disease-related dysfunctions or imbalances. Therefore, these biomarkers are a valuable measure for the evaluation of interventions or therapies as well as for the detection or differentiation of disease patterns (8, 33–36).

### Statistical Analysis

To avoid gaps in the paired samples or replacing them with an artificial value in an additional step, the statistical analysis was performed only with the data of the participants that were available without missing values from M1 to M4 (IGR vs. CON) or to M6 (IGR only). The statistical analysis based on the 32 selected features from both groups was carried out in three major consecutive steps. At first, the measured variables from the feature set were transformed into one joint dimension to eliminate inconsistencies in the data from different origins. After this precondition was met, a factor analysis to further summarize and reduce the feature set in a second step was performed. In the last step the statistical evaluation based on the remaining feature set was completed *via* a multivariate analysis of variance (MANOVA) in a two-way repeated measures design.

#### Standardizing the Variables

In preparation of further statistical analysis the measured variables were standardized. The adapted transformation used the sample mean and standard deviation with reference to the first measurement to compute the z-scores. Since the observed variables were almost the same in both groups at the beginning of the study, the first measurement was set as a common reference point. The standardizing was an important preparation for the subsequent factor analysis where the variable values must come from a uniform dimension. Otherwise, due to the different origins of the variables in physiological and technical terms, the values would have different magnitudes, scales, and units. That imbalance would then lead to incorrect weightings and thus over- or underestimations in the factor analysis.

#### Factor Analysis

With 31 (IGR) and 34 (CON) participants we wanted to further reduce the feature set of still 32 measured variables. This was meant to ensure the statistical accuracy (regarding the ratio of participants/cases to measured variables) and to enhance the statistical power as well as to condense the information from the given variability indices. To achieve this, a factor analysis was performed based on the 32 variables in our feature set. This statistical method searches for representations of correlations in the given data and thus offers the possibility to summarize many variables into fewer factors. The resulting factors define new artificial variables representing a grouping of variables with the same or similar implication. A factor is a linear combination of the respective input variables. It has its own value (score) and holds the information (explained variance) of the variables loading on it. These are weighted according to their information contribution to the factor. Despite a reduced number of variables (the new factor scores), in sum all factors may contain a significant part of the information content of the original data. The mathematical summary of (different) variables into new artificial factor scores does not need to necessarily appear logical and their meaning may be difficult to interpret. However, by using factor analysis, the number of variables could be considerably reduced from 32 measured features to seven factor scores. The factorization seemed logical, and a good interpretation of the individual factors was still given. The factor analysis has been carried out in IBM SPSS Statistics (Version 26). The extraction method was Principal Components Analysis (PCA), the rotation method set to Varimax, the resulting factor scores saved as new variables *via* linear regression method and the input variables consisted of the 32 measured variables at time M1 (over both groups). The following criteria have been considered for an appropriate number of factors: Eigenvalue >1; contribution to explained variance ≥ 5%; explained total variance (cumulative) ≥ 80%.

It was found that the single respiratory variable RESP_meanBPM (average respiratory rate for the entire time series in breaths per minute) had an approximately equal load on several factors. Therefore, a clear assignment to a specific factor was not possible and we decided to define an exclusive factor with only this variable. Subsequently, the factor analysis was performed again with only 31 variables (excluding RESP_meanBPM), resulting in a set of six factors that met the requirements. The total variance explained by these six factors was 84,4% and with addition of the single seventh respiratory factor 87%. The SPSS function calculated the new factor scores using linear regression, but with the contribution of all variables to all factors, no matter how small the actual loadings were. For a stricter differentiation of the factors and a reduction of redundancy in the data, we set two more requirements for factorization:

- variables must have a minimum load of 0.5 on a factor (variables below are discarded because the contribution is too low)
- each variable may only load on one factor (based on its highest contribution)

For this reason, the rotated component matrix, containing the loadings of each variable on each factor, was revised according to these specifications. As this changed the weightings of the linear combinations used to build the factor scores, the coefficients had to be re-estimated. Therefore, another linear regression was performed, but this time with only the specific variables with high loadings on each factor. Using the new coefficients, the factor scores could now be recalculated *via* the sum product of the fixed coefficients and the values of the measured variables. The final factors and their representative meanings are shown in Table 3. A more detailed overview about the six derived factors (excluding factor 7 with only one exclusive respiration variable) and their loading variables are stated in the rotated component matrix in the supplementary materials (Supplementary Table 2).

**Table 3:** Final factors from factor analysis and their representation.

**Factor**	**Description (number of loading variables)**
F1	PWV—PSYS & PDIA variability (10)
F2	HRV—time domain & symbolic dynamics (9)
F3	PWV—correlation & trans-information PSYS & PDIA (5)
F4	HRV—frequency domain (2)
F5	PSYS & PDIA amplitudes (2)
F6	(m)HRJSD—high-resolution joint symbolic dynamics (3)
F7	RESP_meanBPM (1)

#### MANOVA

As described above, the study design was based on two groups which were repeatedly measured at up to six times and numerous medical indices were collected per measurement. A two-way repeated measures MANOVA was performed to model and evaluate the multivariate data with a two-factorial design. This was done using the syntax of the GLM (general linear model) function in IBM SPSS Statistics. The grouping variable GROUP was set as between-subject factor and a new variable TIME was defined as within-subject factor to indicate the number of repetitions of the multivariate data (dependent variables). The alpha level as significance criteria was set to 0.05. The MANOVA was conducted in three different configurations. The first two variants built on each other, and the third explored a further question about the long-term effects of the MBST program. In addition, the estimated marginal means (EMMEANS) were computed as a *post-hoc* test from the MANOVA model. The pairwise comparisons of the EMMEANS provided more detailed insights into which variables potentially indicate differences between the levels of GROUP or TIME. The EMMEANS refer to data estimates based on the model rather than the observed data themselves. However, the observed data formed the model in the first place. In the first run, the seven derived factor scores were taken as dependent variables to examine whether there were statistically significant main effects in GROUP, TIME, or the interaction of both. Following the trace of these results, the statistical analysis continued with a second MANOVA, this time including only the measured PWV features from factor 1 as dependent variables. More specifically, eight out of ten PWV features were used to obtain a more condensed set of features. We have set a threshold of contribution to 0.8 in absolute. Due to the low loading magnitude of 0.6 for PSYS_sdaAMP1 and PDIA_sdaAMP1 compared to the high loadings (> = 0.8) of the other variables on factor 1, these two were excluded from further analysis steps. Moreover, both, long-term and short-term indices were already given by sdAMP and RMSSD, respectively. The averaged 1-min standard deviation windows of the PSYS and PDIA amplitudes as a kind of middle ground between the two standard indices were not considered essential for the evaluation. A third MANOVA configuration was then used to provide a further view of the development of IGR up to the last measurement M6 (1 year follow up). In this case, only the main effect TIME and its pairwise comparisons in EMMEANS have been investigated for IGR exclusively. To address the multiple comparison problem, the Sidak adjustment has been applied. This method was chosen as a good balance between the very conservative Bonferroni correction and the Least Significant Difference (LSD), which has practically no correction for multiple comparisons.

## Results

### IGR and CON at M1 to M4 Regarding 7 Factors

**Figure 1:**
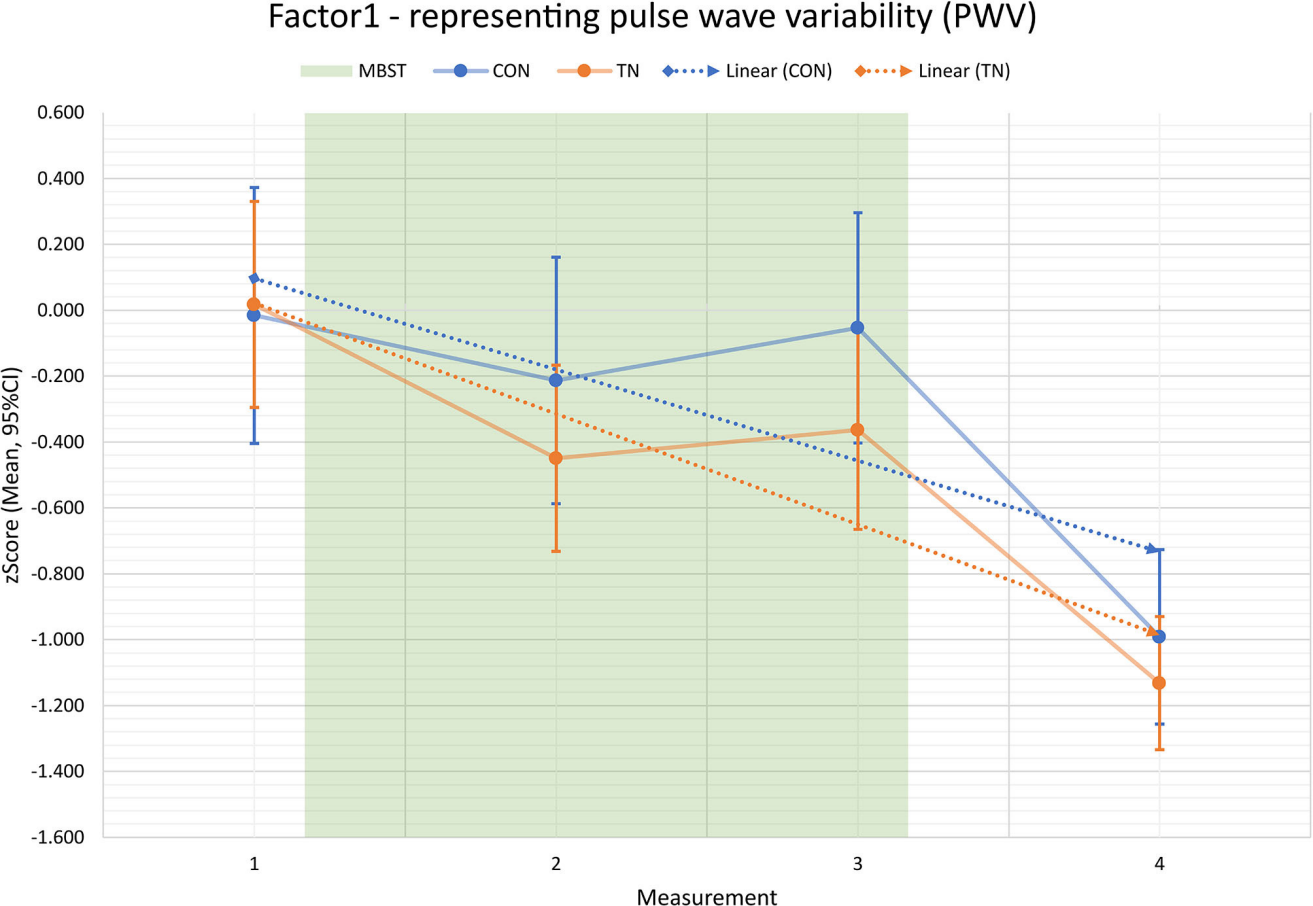
Factor F1 over time—representing pulse wave variability (PWV) progress for intervention group (IGR, orange) and controls (CON, blue) in measurements M1 to M4; M1 to M3: intervention period; M4: 3 months follow-up after intervention; results based on z-values; values show that both groups start from similar conditions, then diverge during Mindfulness-based Student Training (MBST), and the effect persists until 3 months after end of MBST; linear trends (dashed lines) support this impression.

The performed two-way MANOVA examining main GROUP and TIME effects based on the seven factors as dependent variables revealed a statistically significant multivariate TIME effect (Table 4). That indicates a significant change over time across both groups involving the multivariate data. There was no multivariate significant effect for GROUP across all times and for the interaction effect of TIME^*^GROUP. Moreover, in the model-based EMMEANS results, there were also no indications for significant differences between IGR and CON within the four levels of TIME (M1 to M4) regarding multivariate data of the seven factors. The multivariate TIME effect from EMMEANS within the two levels of GROUP (IGR, CON) confirmed the significant changes over time in both groups from the previous results of the observed data (Table 5). However, within the pairwise comparisons of the EMMEANS tables we found evidence for specific significant changes over time within factor F1 (Table 6). While this applied to both groups, changes in CON were always related to measurement M4, whereas changes from M1 to M2 were also evident in IGR. Since M4 was probably strongly influenced by the fact of the summer break at the University, we particularly put our attention on measurements between the intervention period M1 to M3. The comparison between M1 and M3 was never significant for CON, no matter which adjustment was chosen. The same comparison for IGR instead was significant with Fisher's Least Significant Difference (LSD) adjustment, which, though, does not correct for multiple comparisons (not reported here). With the proposed Sidak adjustment, the comparison M1 to M3 for IGR in F1 is no more significant (IGR p ≤ 0.140; CON p ≤ 1.0). We have merely regarded this as a trend. Visual inspection *via* plots of factors and variables supported our assumption of a strong impact of summer break (involving M4) on both groups. These insights seemed to support our findings of altered PWV from the first preliminary study (concerning M1 to M2), and therefore the following analysis focused on the vascular PWV features from factor F1 (see vascular variability progress in Figure 1).

**Table 4:** MANOVA results on 7 factors—multivariate results from TIME (4 levels) BY GROUP (2 levels) analysis with 7 factors as dependent variables.

**Effect**		**Value**	**F**	**Hypothesis df**	**Error df**	**Sig**.	**Partial Eta squared**
**Multivariate tests—TIME and GROUP main effects**							
Between Subjects	Group	0.868	1.239b	7.000	57.000	n.s.	0.132
Within Subjects	Time	0.221	7.214b	21.000	43.000	<0.00	0.779
	Time * Group	0.624	1.234b	21.000	43.000	n.s.	0.376

*Sig. based on Wilks-Lambda at level 0.05. Design, Group; Within Subjects Design, Time. Exact statistic*.

**Table 5:** Results of EMMEANS from MANOVA with 7 factors indicating TIME (4 levels) effect within each GROUP.

**Group [0 = CON, 1 = IGR]**	**Value**	**F**	**Hypothesis df**	**Error df**	**Sig**.	**Partial Eta Squared**
**EMMEANS—Multivariate Tests with TIME effect per GROUP**						
0	0.281	5.244	21.000	43.000	<0.00	0.719
1	0.383	3.294	21.000	43.000	<0.00	0.617

*Each F tests the multivariate simple effects of Time within each level of the other effects shown. These tests are based on the linearly independent pairwise comparisons among the estimated marginal means. Exact statistic using Wilks' lambda at significance level 0.05*.

**Table 6:** Pairwise comparisons from EMMEANS indicating TIME (4 levels) effects between specific measurement times for each GROUP in terms of the measure of factor F1.

**Measure**	**Group [0 = CON, 1 = IGR]**	**Time**		**Mean Difference (I–J)**	**Std. Error**	**Sig**.	**95% Confidence interval for difference**	
		**I**	**J**				**Lower bound**	**Upper bound**
**EMMEANS—Pairwise comparisons of TIMEs per GROUP**								
F1	0	1	2	0.198	0.161	n.s.	−0.241	0.636
			3	0.038	0.158	n.s.	−0.392	0.468
			4	0.976	0.162	<0.00	0.536	1.417
		2	1	−0.198	0.161	n.s.	−0.636	0.241
			3	−0.160	0.128	n.s.	−0.508	0.187
			4	0.779	0.171	<0.00	0.314	1.243
		3	1	−0.038	0.158	n.s.	−0.468	0.392
			2	0.160	0.128	n.s.	−0.187	0.508
			4	0.939	0.154	<0.00	0.520	1.358
		4	1	−0.976	0.162	<0.00	−1.417	−0.536
			2	−0.779	0.171	<0.00	−1.243	−0.314
			3	−0.939	0.154	<0.00	−1.358	−0.520
	1	1	2	0.467	0.169	0.044	0.008	0.926
			3	0.381	0.166	n.s.	−0.069	0.831
			4	1.149	0.170	<0.00	0.688	1.610
		2	1	−0.467	0.169	0.044	−0.926	−0.008
			3	−0.086	0.134	n.s.	−0.450	0.279
			4	0.682	0.179	0.002	0.196	1.169
		3	1	−0.381	0.166	n.s.	−0.831	0.069
			2	0.086	0.134	n.s.	−0.279	0.450
			4	0.768	0.161	<0.00	0.329	1.206
		4	1	−1.149	0.170	<0.00	−1.610	−0.688
			2	−0.682	0.179	0.002	−1.169	−0.196
			3	−0.768	0.161	<0.00	−1.206	−0.329

*Based on estimated marginal means. The mean difference is significant at the 0.05 level. Adjustment for multiple comparisons: Sidak*.

### IGR and CON at M1 to M4 Regarding PWV Variables

The multivariate results of the MANOVA based on eight PWV features from factor F1 as dependent variables showed statistically significant main effects in GROUP and TIME, but not in the interaction of both (Table 7). This means a multivariate difference between both groups across all times as well as differences over time across both groups. Regarding multivariate effects in EMMEANS, there were no significant differences between the groups within the specific four levels of TIME, but a significant effect of TIME within both separate groups (Table 8). This translates to no differentiation of the groups at M1 to M4 across all variables, while both groups vary significantly over time. Further, the significant main effect for GROUP cannot be attributed to a specific measurement time point but arises from the variations across all time points.

**Table 7:** MANOVA results on 8 PWV features—multivariate results from TIME (4 levels) BY GROUP (2 levels) analysis based on 8 pulse wave variability features as dependent variables.

**Effect**		**Value**	**F**	**Hypothesis df**	**Error df**	**Sig**.	**Partial Eta squared**
**Multivariate Test—TIME and GROUP main effects**							
Between subjects	Group	0.741	2.440	8	56	0.024	0.26
Within subjects	Time	0.160	8.767	24	40	<0.00	0.84
	Time * Group	0.600	1.111	24	40	n.s.	0.40

*Sig. based on Wilks–Lambda at level 0.05 Design, Group; Within Subjects Design, Time. Exact statistic*.

**Table 8:** Results of EMMEANS from MANOVA based on 8 PWV features indicating TIME effect within each GROUP.

**Group [0 = CON, 1 = IGR]**	**Value**	**F**	**Hypothesis df**	**Error df**	**Sig**.	**Partial Eta squared**
**EMMEANS—multivariate tests with TIME effect per GROUP**						
0	0.249	5.020	24.000	40.000	<0.00	0.751
1	0.255	4.865	24.000	40.000	<0.00	0.745

*Each F tests the multivariate simple effects of Time within each level of the other effects shown. These tests are based on the linearly independent pairwise comparisons among the estimated marginal means. Exact statistic using Wilks' lambda at significance level 0.05*.

Further, we considered the individual pairwise comparisons among the specific eight PWV variables and found no evidence for significant differences between IGR and CON. However, a decreasing trend in *p*-values was observed, indicating a slightly greater potential for differentiation over the duration of the intervention, particularly in M2 and M3 compared to baseline in M1.

**Figure 2:**
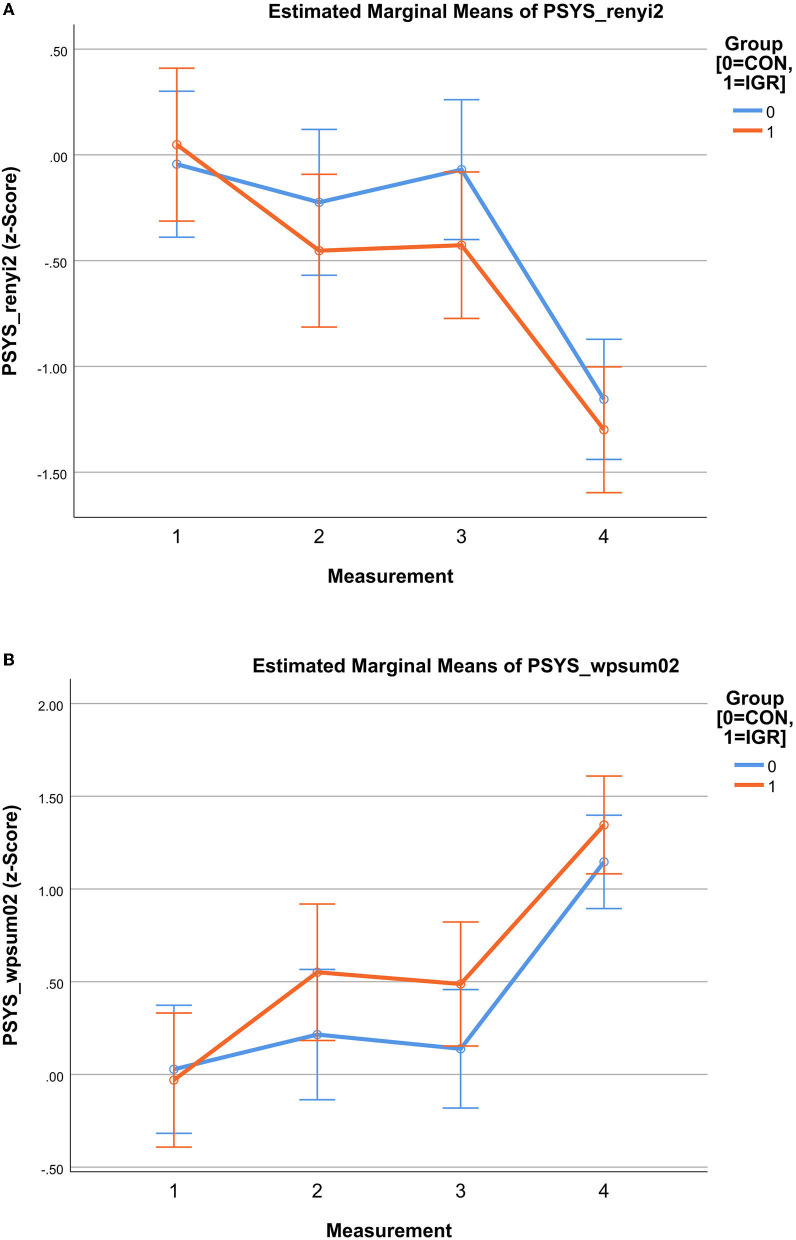
Mindfulness-based Student Training (MBST)—results of Estimated Marginal Means of pulse wave variability (PWV) showing the progress for intervention group (IGR) and controls (CON) in measurements M1 to M4; M1 to M3: intervention period; M4: 3 months follow-up after intervention; results based on z-values **(A)** PSYS_renyi2 as PWV estimate; Rényi2 entropy is a measure for complexity; cardiovascular variability starts similar (M1), then diverges with lower levels for IGR until MBST end (M3) and still remains lower in follow-up (M4); **(B)** PSYS_wpsum02 as PWV estimate; wpsum02 is a measure for decreased variability (higher values means lower variability); cardiovascular variability starts similar (M1), then diverges with higher levels for IGR until MBST end (M3) and still remains higher in follow-up (M4).

After direct comparisons between the groups, pairwise longitudinal comparisons were examined regarding changes over time within both groups. First, significant differences were again seen in relation to M4. This applied to all eight PWV features and both groups, just as the results from the analysis of factor F1 had already shown. In the second, however, significant changes in M1 to M2 or M3 were found exclusively for the intervention group. In detail, PSYS_renyi2 and PSYS_wpsum02 (see Figure 2) revealed statistically significant changes within IGR from M1 to M2 (PSYS_renyi2 *p* ≤ 0.031; PSYS_wpsum02 *p* ≤ 0.007) as well as from M1 to M3 (PSYS_renyi2 *p* ≤ 0.041; PSYS_wpsum02 *p* ≤ 0.018). The results of the other PWV features supported this outcome of exclusive changes for IGR only as trends. The comparison from M2 to M3 was not significant indicating a constant level. Except for M4, we did not notice any significant changes within the progress of CON.

### IGR at M1 to M6 Regarding PWV Variables

**Figure 3:**
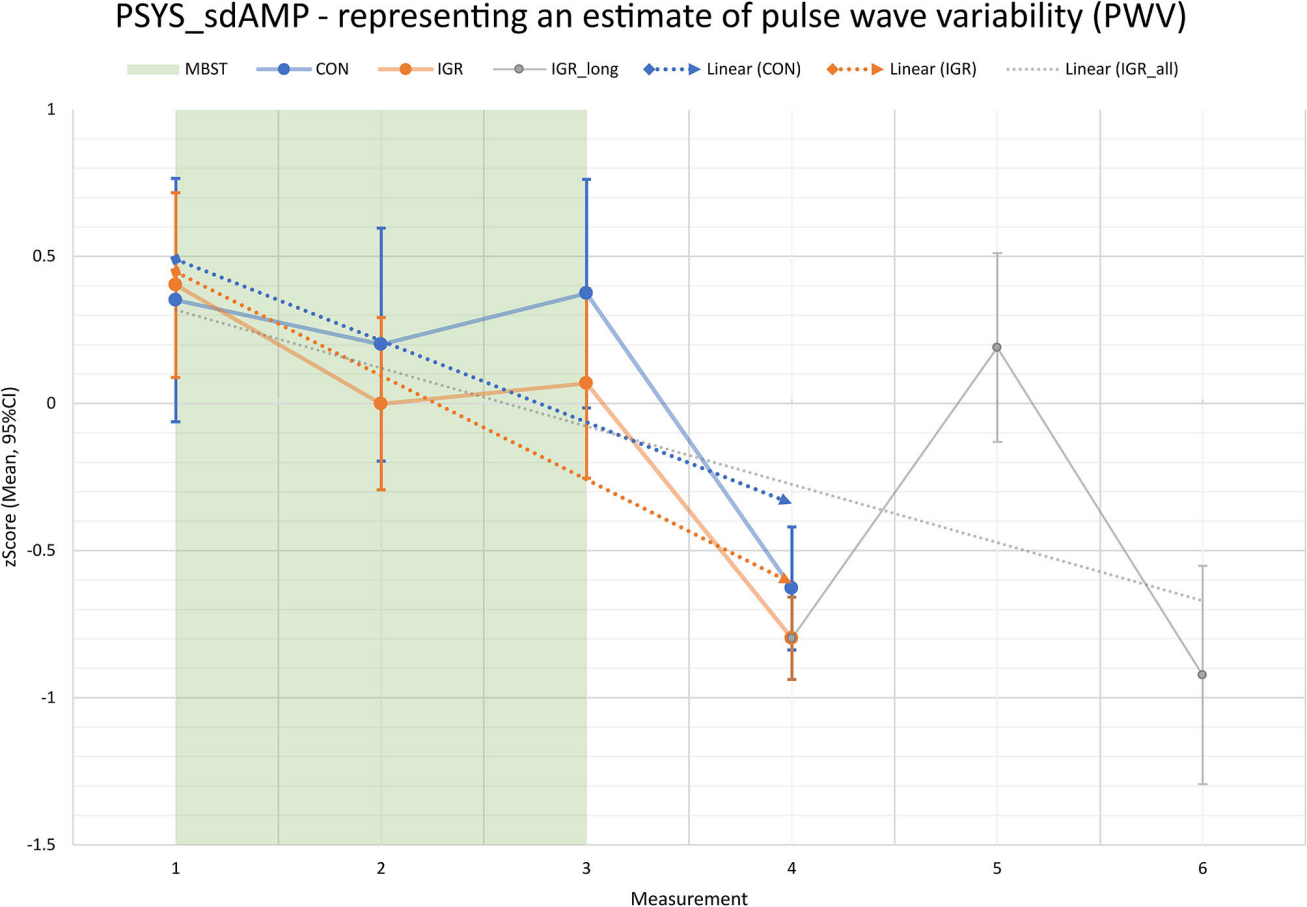
Pulse wave variability (PWV, represented by PSYS_sdAMP estimate) progress for intervention group (IGR) and controls (CON) across entire study period (measurements M1 to M6); M1 to M3: intervention period; M4 to M6: 3, 6, and 12 months follow-up after intervention; direct comparison between IGR and CON until M4; measurements M5 and M6 show exclusive longitudinal outlook for IGR (data from CON not available); PSYS_sdAMP as standard deviation of the systolic amplitude series from the pulse wave signal is a measure for vascular variability.

First, the multivariate analysis based on the observed data revealed a statistically significant TIME effect (Table 9). But second, there was no statistically significant TIME effect with respect to the multivariate data from the model. However, the pairwise comparisons showed some comparable results to the previous longitudinal comparisons within IGR on the eight PWV features. Most comparisons involving M4 or M6 point to significant differences related to these times. Further, the systolic PWV features PSYS_sdAMP, PSYS_renyi2 and PSYS_wpsum02 revealed significant alterations after 8 weeks of MBST (M1 to M2). However, this time there was no evidence of significant changes from M1 to M3 or M2 to M3 either. Visualization by plots illustrated the progression with decreasing PWV after 8 weeks of MBST (M1 to M2), followed by a slight increase in variability at the end of MBST (M3) and just before the exams. Yet, it remained below the level of M1. Then there was a notable drop 3 months after the end of the intervention and after summer break (M4). After another 3 months and before the next exams (M5), there was again a major increase to near M1 level. Finally, one year after the end of MBST and during the subsequent semester (M6), the PWV showed another strong reduction toward the level of M4. To provide an entire overview (Figure 3), we have integrated the mixed data into one chart to illustrate the progress of IGR and CON up to 3 months after MBST (M4) as well as the long-term prospect for the intervention participants up to one year in follow-up (M6). Underlying data were based on complete participant strength for both groups through M4 and then continued with reduced participant numbers in IGR through M6. Linear trendlines support the visualization of the progress. Particularly, the graph shows both the built-up difference in vascular variability (represented by PSYS_sdAMP) between intervention group and controls due to significant MBST induced alterations, and the continuing trend of former MBST participants up to 1 year after course end.

**Table 9:** MANOVA results—multivariate results for intervention group from TIME (6 levels) effect analysis based on 8 pulse wave variability features as dependent variables.

**Within subjects effect**		**Value**	**F**	**Hypothesis df**	**Error df**	**Sig**.	**Partial Eta squared**
**Multivariate test—TIME main effect**							
Time	Wilks' Lambda	0.162	5.278	40	408.172	<0.00	0.305

*Within Subjects Design: Time. Tests are based on averaged variables. The statistic is an upper bound on F that yields a lower bound on the significance level*.

**Figure 4:**
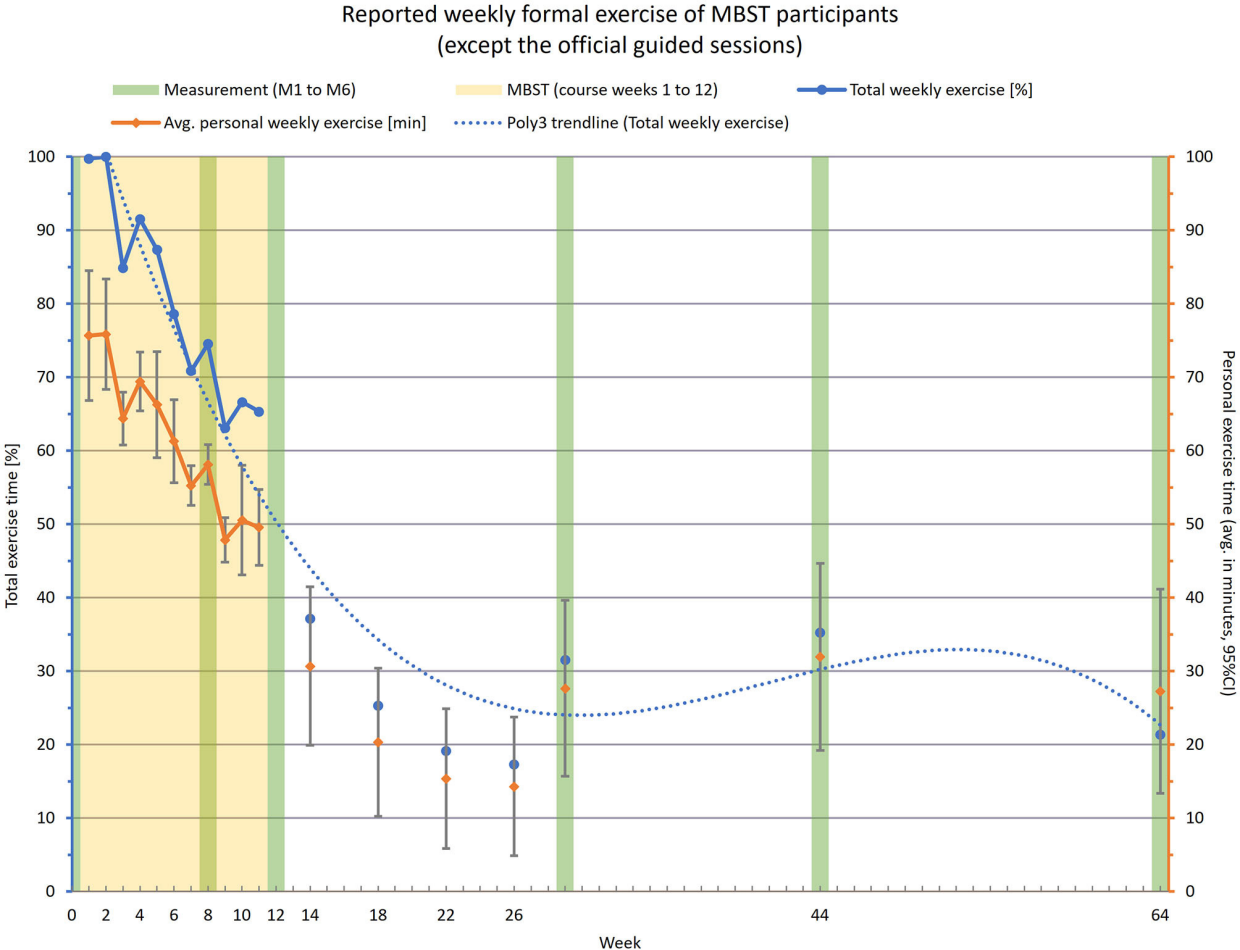
Chart of reported weekly formal exercise performed by intervention participants; excluding the official training sessions; left axis/blue data—total weekly exercise time as cumulative effort of all participants in %; right axis/orange data—individual weekly exercise time as average effort per participant in minutes; measurements took place at weeks W0 (pre-intervention), W8, W12 (end of intervention), W29 (3 months after end), W44 (6 months after end) and W64 (1 year after end).

In addition, information was gathered on participants' weekly formal exercise time at home. The weekly officially guided sessions during MBST period were not included in this summary. The overview in Figure 4 illustrates the weekly total exercise across all participants as well as the individual averaged exercise time. During the 12-week MBST period the weekly protocolled total exercise time decreased to about 65% at the end of the intervention compared to the first week. The individual time spent for home practice decreased from 75 min after start to 50 min per week at the end of MBST. The surveyed exercise times with increasing intervals in the follow-up showed a further decrease in the months after the end of the course, but also a slight increase at the measurement dates M4 to M6. The impressions from the short personal interviews showed that most of the former participants had either completely abandoned the exercises they had learned or had increasingly integrated them into their everyday lives. A moderate intermediate path was rarely reported. Based on this information with progressively more divergent engagement in continuation of exercise practice, the results for IGR until M6 are as mentioned only a vague prospect.

## Discussion

In the present longitudinal study, we examined the effects of a 12-weeks Mindfulness-Based Student Training on the autonomic regulation of the intervention participants. Among detailed methodological elements, this student-tailored intervention was primarily intended to strengthen stress management as well as self- and emotion regulation for coping with everyday University life. In this study, the potential beneficial physiological effects were assessed by analyzing HRV, PWV, and respiratory indices. The comparison included the evaluation of a passive control group to estimate group and time effects between and within both groups. The statistical analysis was based on two major steps: factor analysis for summarizing and reducing the rich feature set as well as MANOVA with additional *post-hoc* testing for multivariate and univariate time and group effects.

### Comparison of IGR and CON Up to Three Months Follow-Up

In summary we can admit a positive impact on vascular functions for the MBST intervention group. The first step utilizing a MANOVA with components of a factor analysis estimating HRV, PWV and respiration activity pointed to statistical differences within the PWV. In the second step, the pairwise comparisons gave evidence of significantly reduced PWV after 12 weeks of intervention exclusively within the MBST participants. It can be seen that both groups follow the same trend across the measurement points, though to different degrees. This correlates with the study situation during the semester and is consistent with our perceptions on campus and feedback from participants. This is reflected in slight stress at the beginning of the semester due to new courses and requirements (M1), less stress in the middle of the semester (M2), high stress at exam time at the end of the semester (M3), and significant relaxation at/after semester break (M4). Nevertheless, the MBST with the taught methods for stress management seems to have an effect, as the described trend of the stress effect toward the measurement points is lower in the intervention group and/or a stronger resistance or adaptation can be observed here. Although both groups started at almost the same baseline, the difference in PWV between IGR and CON grew over the intervention duration. Even in a phase of elevated stress due to exams at the University at the end of the intervention, the observed effect persisted. Moreover, the difference was reduced but still observed in the 3-month follow up after end of MBST. Although the effects of summer break on both groups were much greater at this time, resulting in a decreased PWV and less stress for both groups, there was still a measurable difference in favor of the intervention group. Apart from the vascular features estimated by the PWV, we could not identify evidence of significant changes in HRV or breathing rate, neither over time nor between the groups.

The main finding from this study is that especially the vascular component (surrogate to systolic blood pressure) is positively modeled by MBST. In particular, the systolic vascular variability parameters showed significant differences in non-linear dynamics (renyi2, wpsum02) and in the time domain (sdAMP). This reduced PWV can be interpreted as clear signs of stress reduction, which is discussed in more detail below. Additionally, the trend of the other but non-significant systolic as well as diastolic variability parameters supports this finding.

Short-term blood pressure variability or short-term pulse wave variability on a beat-to-beat basis, as linear and non-linear markers of the (cardio-) vascular system, have not, to our knowledge, been investigated in any other study of mindfulness training in University students to date. A few systematic reviews and meta-analyses suggested that mindfulness-based interventions can reduce blood pressure (37). These studies mostly showed only a reduction in systolic and diastolic blood pressure in people with hypertension (38) or elevated blood pressure. Increased stress levels are associated with coronary heart disease incidence, increased mortality, and poorer health in patients with acute myocardial infarction or hypertension, independent of traditional risk factors. This is a consequence of an altered (cardio-) vascular situation. Long term variability in blood pressure is associated with cardiovascular and mortality outcomes, over and above the effect of mean blood pressure (39). Limited data for mid-term and short-term variability showed similar associations (40). Blood pressure is a continuous, not a static, variable. Individuals exhibiting similar clinic or home blood pressure can differ considerably with respect to their average day and nighttime values, beat-by-beat blood pressure variation during wakefulness and sleep, responses to mental and physical stimuli, and intersession and seasonal variation (41).

High blood pressure variability may be a predictor for the risk of dementia or cognitive impairment. The relative contribution of variability in blood pressure exceeded that of mean blood pressure (42). It could be shown that blood pressure variability has significant association with the quality of life in a small group of mildly hypertensive patients (43). It was concluded that an improvement in quality of life may lead to a favorable change (reduction) in blood pressure variability. Accordingly, the reduction in vascular variability by MBST intervention found in our study may be a clear response in this direction, toward more relaxed, and thus healthier, regulation.

The brain controls the heart directly through the sympathetic and parasympathetic branches of the autonomic nervous system. Cardiac function can be profoundly altered by the reflex activation of cardiac autonomic nerves in response to inputs from baro-, chemo-, nasopharyngeal and other receptors as well as by central autonomic commands, including those associated with stress, physical activity, arousal, and sleep (44). Among other effects, cardiac functions are altered, for example, by disinhibition of subregions of the medial pre-frontal cortex and adjacent regions (45). The autonomic outflow to the heart is regulated by a central autonomic network of interconnected brain structures, which includes the medial pre-frontal cortex and insular cortex, the amygdala and the bed nucleus of the stria terminalis, the lateral region of the hypothalamus and the paraventricular nucleus and dorsomedial hypothalamic nucleus, the periaqueductal gray matter of the midbrain, the parabrachial Kölliker–Fuse region of the lateral pons, as well as several regions of the medulla (44). Thus, increased mean arterial pressure has been shown to correlate with the response of several cortical and subcortical brain regions thought to be responsible for controlling cardiovascular responses to behavioral stressors (46). These regions include the perigenual and middle anterior cingulate cortex, medial and lateral regions of the pre-frontal cortex, the insula, the periaqueductal gray mat, and the cerebellum. We hypothesize that the reduction in vascular variability found by stress reduction originates in these brain regions and is ultimately mediated by the baroreflex. A study by Sevinc et al. (47) also points in this direction, showing that mindfulness training improves cognition and strengthens intrinsic connectivity between the hippocampus and posteromedial cortex in healthy older adults.

### Exclusive Long-Term Outlook for IGR Up to One Year Follow-Up

Regarding the longitudinal progress exclusively for the intervention group with up to 1 year follow-up after end of MBST, we can confirm the results and trend of reduced vascular variability. Furthermore, before the examens at the end of the next semester (6 months follow-up), the PWV of the former participants raised to nearly pre-intervention level and though higher than at the end of MBST. Nevertheless, at 1-year follow-up, another strong decrease in PWV was observed, indicating a positive long-term effect. The linear trend function for the progress of PWV estimates from the (former) MBST participants supports the assessment of a sustainable positive effect.

### Limitations

When considering all the data and results, some limitations of the study should be noted. Since this training was specifically designed for and conducted by University students, the conclusions are not necessarily valid for the general public. In addition, it must be mentioned that most of the participants were students of social sciences (besides students of economics and engineering) and that the gender ratio was biased in favor of women. This double imbalance, in combination with possible affinities or sensitivities of participant composition, may yield specific influences on the effects of mind-body interventions. The registration process was quasi-randomized (first come first serve due to knowledge of recruitment advertising at University) but not strictly following the procedures of an RCT. Also, the influence of some meta-information as cofactors such as age, gender, sports activities, and meditation or yoga experience has not been investigated. These might affect the impact of MBST training on the participants and thus the results of the study. However, possible influencing factors due to certain diseases and conditions were already excluded at the time of registration due to eligibility requirements. Finally, the outlook on the progress of former intervention participants up to 1 year in follow-up can only serve as an orientation. There was no record of controls for the times beyond the 3 months follow-up which prevents a detailed between-groups comparison regarding long-term effects at 6 months and 1 year after end of intervention. Additionally, many factors in daily life of the former participants might have changed over time (such as the degree of integration of MBST elements in daily routine). The insights from the participants' responses about the (in average) decreasing but also widely diverging extent of reported exercise at home during and after the MBST intervention may emphasize this point.

Stress reduction, as mentioned earlier, is of great personal as well as public health importance in a wide variety of areas. For young people and students, various approaches are being taken in this regard (48), such as the use of yoga (49), meditation (50), biofeedback procedures (51, 52), and even different mindfulness-based interventions (37, 53, 54).

The use of questionnaires in the detection and/or assessment of stress or stress levels has been or is the main approach. Though, there is a growing interest in identifying the physiological parameters representing autonomic regulation that correlate with the detection of stress as well as the reduction of stress levels. In particular, these include electrodermal activity, heart rate, heart rate variability, respiration, blood pressure, pulse, and vascular variability (32, 37, 50, 51, 55). Stress affects the cardio-vascular system, which can be detected by parameters of the pulse curve (32) or blood pressure (56).

The physiological response to an acute stressor is usually much stronger than to a slow increase in or persistence of an elevated stress level (53, 57–59). This effect is particularly evident in studies examining the response of autonomic regulation in the form of HRV and respiration. In contrast, the parameters of the vascular system (*via* PWV) seem to be more sensitive regarding the latter effect, according to our experience.

The most important finding of this study is that MBST in particular has a positive effect on certain parameters of the cardio-vascular system not only acutely but also persistently, which can be considered as a promising marker of sustained stress level reduction in students.

### Implications of the Study

The impact analysis of mindfulness training has already been the subject of numerous scientific studies in the past. The article by Haase and Lautenschläger (60) is specifically dedicated to the formats designed and implemented within the framework of the Thuringian Model Mindful Universities. It asks in particular to what extent the MBST can have an effect on mindfulness, stress perception, internet addiction and well-being of students. Standardized instruments were used to survey 197 participants in 12 MBST courses between April 2018 and July 2019. The results show that significant changes in mindfulness, subjective stress levels, and compulsive or unhealthy Internet use among participants can be identified from the comparison of measurements at the beginning and end of the event cycles. Even though the analysis of Haase and Lautenschläger (60) has limitations resulting from the exploratory study design, especially the lack of a control group, and even though causal relationships cannot be derived with the chosen research design, it underlines the potential of MBST for students with regard to stress management and the improvement of daily life. Their study complements the present study and points to the effectiveness of MBST also with regard to the life-serving use of digital media.

The downside of the multitude of new digital possibilities and constant accessibility is feeling permanently driven. What can be observed is a dissolution of boundaries and new dangers of addiction (61, 62). In this way, digitalization also has a considerable impact on the mental landscape of students and also on their physical health. At the same time, the observed excessive use of smartphones and social media is a risk factor for academic performance (63). Here, according to the studies, MBST has the potential to counteract at various levels.

This is especially true in light of a worrisome trend. Despite the multitude of social media, studies observe a growing loneliness (e.g., Spitzer (64) p.117ff). Sherry Turkle, Professor of Science, Technology and Society at the Massachusetts Institute of Technology (MIT) puts it in the following words: “We slip into thinking that always being connected is going to make us less lonely. But we are at risk because it is actually the reverse: If we are unable to be alone, we will be more lonely. And if we don't teach our children to be alone, they will only know how to be lonely.” (65).

### Conclusion

The results of this MBST evaluation study, based on analysis of key indices of autonomic regulation represented by PWV, HRV, and respiratory activity, show a sustained positive effect toward stress reduction. Participants primarily benefit from reduced vascular variability, reflecting improved autonomic function through reduction of PWV. This effect can already be seen after 8 weeks of intervention, confirming the results of our first preliminary study. Moreover, it persists for 3 months after the end of the 12-week intervention phase. Furthermore, the results of the long-term analysis up to 1 year after the end of MBST indicate a long-lasting effect among former participants. This effect could be further enhanced by continued practice and integration of the taught exercises as well as the concepts into everyday life. In perspective, our results could provide guidance for future applications to monitor individual stress levels and intervention progress through wearables with pulse wave analysis.

Due to the positive physiological effect, the MBST intervention can make a meaningful and sustainable contribution to stress reduction and thus to health prevention among students.

## Data Availability Statement

The data of this study are not publicly available because the study has not yet been completed, and further evaluations are currently in progress. However, the data are available on request from the corresponding author.

## Ethics Statement

The studies involving human participants were reviewed and approved by Institutional Ethics Commission of the University Hospital Jena (4509-08/15). The patients/participants provided their written informed consent to participate in this study.

## Author Contributions

Conceptualization: AV, MB, and MS. Methodology, validation, and formal analysis: AV, MB, and MW. Software, visualization, writing—original draft preparation, and project administration: AV and MB. Investigation: MB, BL, and RA. Resources: AV, MB, BL, and RA. Data curation: MB. Writing—review and editing: AV, MB, MW, MS, and GS. Supervision: AV. Funding acquisition: AV and MS. All authors have read and agreed to the published version of the manuscript.

## Funding

The proposed study was funded by the health insurance AOK PLUS as a member of The Federal Association of AOK-Bundesverband GbR in Germany. An exertion of influence by the funder did not take place. The funder was not involved in the study design, collection, analysis, interpretation of data, the writing of this article or the decision to submit it for publication.

## Conflict of Interest

The authors declare that the research was conducted in the absence of any commercial or financial relationships that could be construed as a potential conflict of interest.

## Publisher's Note

All claims expressed in this article are solely those of the authors and do not necessarily represent those of their affiliated organizations, or those of the publisher, the editors and the reviewers. Any product that may be evaluated in this article, or claim that may be made by its manufacturer, is not guaranteed or endorsed by the publisher.
