# Supplementary material for: Mindfulness-Based Student Training Improves Vascular Variability Associated With Sustained Reductions in Physiological Stress Response
Source: Front Public Health. 2022 Jul 18;10:863671. doi: 10.3389/fpubh.2022.863671 (PMC9340219; doi:10.3389/fpubh.2022.863671)
Supplement: Supplementary file 1 [file Table_1.pdf]

Supplementary Material

Supplementary Table 1. Listing and brief description of included measured variables for statistical analysis.

#	Variable	Description
1	RESP_meanBPM	mean respiratory rate for the entire time series in [breaths per minute]
2	HRV_meanNN	mean value of all NN-intervals in [ms]
3	HRV_sdNN	standard deviation (sd) of all NN-intervals in [ms]
4	HRV_sdaNN1	standard deviation of the averages of NN-intervals in all 1-minute segments in [ms]
5	HRV_rmssd	square root of the mean squared differences of successive NN-intervals in [ms]
6	HRV_pNN50	proportion derived by dividing the number of interval differences of successive NN-intervals greater than 50 ms by the total number of NN-intervals
7	HRV_renyi2	Rényi entropy of the histogram of NN-intervals with (order) alpha=2
8	HRV_LFHF	ratio of low frequency (0.04-0.15Hz) and high frequency (0.15-0.4Hz) power spectra estimates
9	HRV_LFN	normalized LF, described by $LFN = \frac{LF}{(LF+HF)}$
10	HRV_wpsum02	relative portion (sum/total) of words consisting only of the symbols ‘0’ and ‘2’,<br><br>measure for decreased HRV
11	HRV_wpsum13	relative portion (sum/total) of words consisting only of the symbols ‘1’ and ‘3’,

		measure for increased HRV
12	PSYS_meanAMP	mean systolic maximum (PSYS) amplitude values from PPG pulse wave
13	PSYS_sdAMP	standard deviation of all PSYS amplitude values
14	PSYS_sdaAMP1	standard deviation of the averages of PSYS amplitude values in all 1-minute segments
15	PSYS_rmssd	square root of the mean squared differences of successive PSYS amplitude values
16	PSYS_renyi2	Rényi entropy of the histogram of PSYS amplitude values with (order) alpha=2
17	PSYS_wpsum02	relative portion (sum/total) of words consisting only of the symbols ‘0’ and ‘2’,<br><br>measure for decreased PWV
18	PDIA_meanAMP	mean diastolic minimum (PDIA) amplitude values from PPG pulse wave
19	PDIA_sdAMP	standard deviation of all PDIA amplitude values
20	PDIA_sdaAMP1	standard deviation of the averages of PDIA amplitude values in all 1-minute segments
21	PDIA_rmssd	square root of the mean squared differences of successive PDIA amplitude values
22	PDIA_renyi2	Rényi entropy of the histogram of PDIA amplitude values with (order) alpha=2
23	PDIA_wpsum02	relative portion (sum/total) of words consisting only of the symbols ‘0’ and ‘2’,<br><br>measure for decreased PWV
24	CORTI_a31NN	slope of the auto-transinformation function (ATI) from the maximum of ATI (tau=0) to the value tau=2 (NN-intervals)

25	CORTI_a31PSYS	slope of the auto-transinformation function (ATI) from the maximum of ATI (tau=0) to the value tau=2 (PSYS)
26	CORTI_a31PDIA	slope of the auto-transinformation function (ATI) from the maximum of ATI (tau=0) to the value tau=2 (PDIA)
27	CORTI_a31NNcor	slope of the auto-correlation function (ACOR) from the maximum of ACOR (tau=0) to the value tau=2 (NN-intervals)
28	CORTI_a31PSYScor	slope of the auto-correlation function (ACOR) from the maximum of ACOR (tau=0) to the value tau=2 (PSYS)
29	CORTI_a31PDIAcor	slope of the auto-correlation function (ACOR) from the maximum of ACOR (tau=0) to the value tau=2 (PDIA)
30	HRJSDrenyi2_NNPSYS	Rényi entropy of the HRJSD interactions between NN and PSYS series with (order) alpha=2
31	HRJSDrenyi2_NNPDIA	Rényi entropy of the HRJSD interactions between NN and PDIA series with (order) alpha=2
32	mHRJSDrenyi2	Rényi entropy of the multivariate HRJSD interactions between NN, PSYS, and PDIA series with (order) alpha=2

HRV – heart rate variability; NN – normal-to-normal heart beat intervals; PSYS/PDIA – systolic maximum/diastolic minimum amplitude series from PPG (photoplethysmogram) pulse wave; PWV – pulse wave variability; CORTI – (auto-) correlation and (auto-) transinformation analysis as measure for complexity (similarity in mutual information) and variability; HRJSD – high-resolution joint symbolic dynamics as measure for complexity (in symbolic dynamics) and variability
