# Supplementary material for: Mindfulness-Based Student Training Improves Vascular Variability Associated With Sustained Reductions in Physiological Stress Response
Source: Front Public Health. 2022 Jul 18;10:863671. doi: 10.3389/fpubh.2022.863671 (PMC9340219; doi:10.3389/fpubh.2022.863671)
Supplement: Supplementary file 2 [file Table_2.pdf]

# *Supplementary Material*

**Supplementary Table 2. Rotated Component Matrix illustrating the six factors and their loading variables (with magnitudes) taken from the factor analysis of reference measurement M1 (excluding respiration variable in exclusive factor 7).**

| Rotated Component Matrix | | | | | | |
|---|---|---|---|---|---|---|
| Variable | Factor | | | | | |
| | 1 | 2 | 3 | 4 | 5 | 6 |
| M1_HRV_meanNN | | 0.5 | | | | |
| M1_HRV_sdNN | | 0.9 | | | | |
| M1_HRV_sdaNN1 | | 0.5 | | | | |
| M1_HRV_rmssd | | 0.9 | | | | |
| M1_HRV_pNN50 | | 0.9 | | | | |
| M1_HRV_renyi2 | | 1.0 | | | | |
| M1_HRV_LFHF | | | | 0.9 | | |
| M1_HRV_LFN | | | | 0.9 | | |
| M1_HRV_wpsum02 | | -0.9 | | | | |
| M1_HRV_wpsum13 | | 0.6 | | | | |
| M1_PSYS_meanAMP | | | | | 0.8 | |
| M1_PSYS_sdAMP | 0.96 | | | | | |
| M1_PSYS_sdaAMP1 | 0.64 | | | | | |
| M1_PSYS_rmssd | 0.91 | | | | | |

| | | | | | | |
|---|---|---|---|---|---|---|
| M1_PSYS_renyi2 | 0.97 | | | | | |
| M1_PSYS_wpsum02 | -0.92 | | | | | |
| M1_PDIA_meanAMP | | | | | -0.8 | |
| M1_PDIA_sdAMP | 0.94 | | | | | |
| M1_PDIA_sdaAMP1 | 0.63 | | | | | |
| M1_PDIA_rmssd | 0.85 | | | | | |
| M1_PDIA_renyi2 | 0.93 | | | | | |
| M1_PDIA_wpsum02 | -0.80 | | | | | |
| M1_CORTI_a31RR | | -0.7 | | | | |
| M1_CORTI_a31PSYS | | | 0.6 | | | |
| M1_CORTI_a31PDIA | | | 0.8 | | | |
| M1_CORTI_a31RRcor | | | 0.5 | | | |
| M1_CORTI_a31PSYScor | | | 0.9 | | | |
| M1_CORTI_a31PDIAcor | | | 0.9 | | | |
| M1_HRJSDrenyi2_BBIPSYS | | | | | | 0.7 |
| M1_HRJSDrenyi2_BBIPDIA | | | | | | 0.8 |
| M1_mHRJSDrenyi2 | | | | | | 0.8 |

Extraction Method: Principal Component Analysis.
Rotation Method: Varimax with Kaiser Normalization.
Rotation converged in 7 iterations.
