# Supplementary material for: Mindfulness-Based Student Training Improves Vascular Variability Associated With Sustained Reductions in Physiological Stress Response
Source: Front Public Health. 2022 Jul 18;10:863671. doi: 10.3389/fpubh.2022.863671 (PMC9340219; doi:10.3389/fpubh.2022.863671)
Supplement: Supplementary file 3 [file Image_1.pdf]

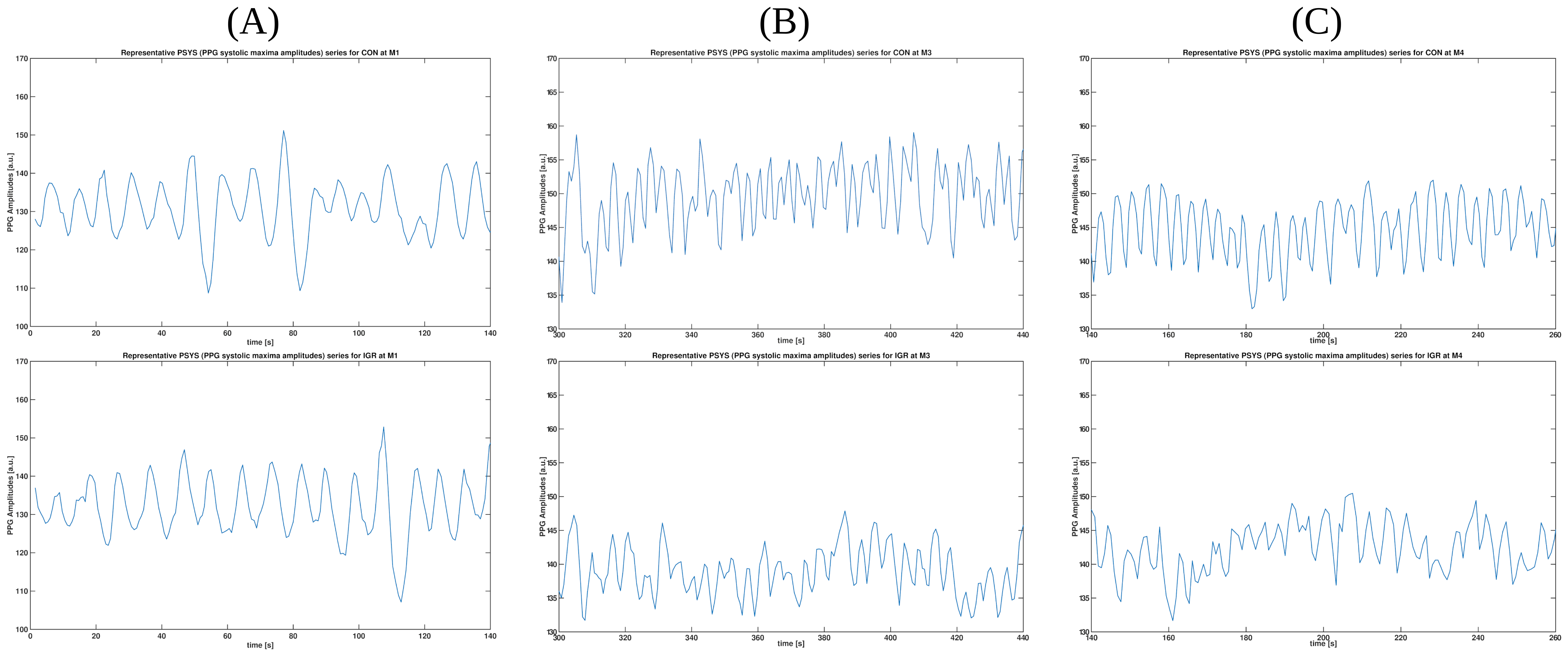

Supplementary Figure 1. Representative visualizations of PSYS (systolic maximum amplitudes from raw pulse wave photoplethysmography/PPG curves) series with typical (in terms of their standard deviation/SD) curves for individual participants in the control (CON, at top) and intervention (IGR, at bottom) groups at three measurement times (M1, M3 and M4), respectively. These graphs represent signal examples that were the basis for all further feature calculations. Regarding the dynamics (here: the fluctuations in amplitude height as well as the frequency of these fluctuations), it can be seen that **(A)** at M1 (before the intervention) the signals appear almost the same for both groups; **(B)** this changes at M3 (end of intervention), where the CON (top) example has a higher dynamic than the IGR (bottom) representative; **(C)** the difference remains at M4 (follow-up, after the semester break).
